# Transformation and Tumorigenicity Testing of Simian Cell Lines and Evaluation of *Poliovirus* Replication

**DOI:** 10.1371/journal.pone.0169391

**Published:** 2017-01-03

**Authors:** Silvia Dotti, Tina Lombardo, Riccardo Villa, Andrea Cacciamali, Cinzia Zanotti, Nadia Andrea Andreani, Stefano Cinotti, Maura Ferrari

**Affiliations:** Istituto Zooprofilattico Sperimentale della Lombardia e dell’Emilia Romagna, Brescia, Italy; Swedish Neuroscience Institute, UNITED STATES

## Abstract

The key role of cell cultures in different scientific fields is worldwide recognized, both as *in vitro* research models alternative to laboratory animals and substrates for biological production. However, many safety concerns rise from the use of animal/human cell lines that may be tumorigenic, leading to potential adverse contaminations in cell-derived biologicals.

In order to evaluate the suitability of 13 different cell lines for *Poliovirus* vaccine production, safety and quality, *in vitro/in vivo* tumorigenicity and *Poliovirus* propagation properties were evaluated.

Our results revealed that non-human primate cell lines CYNOM-K1, FRhK-4, 4MBr-5 and 4647 are free of tumorigenic features and represent highly susceptible substrates for attenuated Sabin *Poliovirus* strains. In particular, FRhK-4 and 4647 cell lines are characterized by a higher *in vitro* replication, resulting indicated for the use in large-scale production field.

## Introduction

Poliomyelitis is a highly contagious disease caused by a virus of the *Enterovirus* genus, belonging to the *Picornaviridae* family, known as *Poliovirus* and composed by a 7,500 nucleotides (+) single stranded RNA molecule [1,2]. Three different serotypes of wild *Poliovirus* were identified and classified as type 1, type 2 and type 3 [3]. No specific therapy is available against the virus, but effective inactivated and attenuated vaccines are essential to prevent the disease. Since the development of the first vaccines by Salk in 1955 and Sabin in 1960 [4,5], *Poliovirus* study greatly improved, taking advantage of cell cultures to isolate the virus from infected people [6,7], microcarrier technology [8,9] and simian cell lines for large-scale production of infected cells for vaccine manufacture [10–14].

Immortalization of animal and human cells, derived from primary cell cultures, is a phenomenon mainly due to genetic mutations or infections by oncogenic viruses, which can result in the appearance of transformed features and tumorigenic properties. Furthermore, cells can undergo several modifications during *in vitro* cultivation, resulting in the appearance of novel biochemical, biological and genetic characteristics that differ from primary or diploid cell ones. This represents an important issue in order to establish the biosafety of the cell lines used as substrates and to monitor the possible transmission of animal pathogens to human recipients [15].

Among continuous cell lines, the human HeLa cell line, naturally contaminated by human *Papillomavirus*, revolutionized the study of *Poliovirus* biology. On the other hand, Vero cells, widely used in *Poliovirus* vaccine manufacturing, became immortalized through a spontaneous, unknown process and they acquired tumorigenic properties with increasing *in vitro* passage levels [16–18]. Moreover, recent studies have demonstrated that the *in vitro* establishment of two African green monkey kidney derived cell lines, named BS-C-1 and CV-1, gave rise to transformed colonies and tumor formation in the rat model [18–20].

The aim of this research was to identify cell lines free of any transformation ability and tumorigenicity, suitable for *Poliovirus* vaccine production. In this respect, thirteen simian cell lines have been screened *in vitro* and *in vivo* for transformation and tumorigenicity features and their permissiveness to *Poliovirus* infection investigated, in comparison with other well-established substrates.

## Materials and Methods

### Cell lines

All the investigated simian cell lines reported in Table 1 were stored at the Italian Biobank of Veterinary Resources of IZSLER, the OIE Collaborating Centre for Veterinary Biological Biobank (Brescia, Italy; www.ibvr.org) and are available upon request. All these are continuous, spontaneously immortalized cell lines, exception made for CYNOM-K1, CV-1 (finite cell lines) and 4MBr-5 (EFG-dependent line). The investigations were performed at the passages indicated.

**Table 1:** Cell lines used in the study.

	Cell line	Species and Tissue Origin	Original source and catalogue number	IZSLER Biobank code	Passage
Tested cell lines	BGMK	Buffalo Green Monkey Kidney	Flow Laboratories	BS CL 07	95
	BS-C-1	African Green Monkey Kidney	ATCC° CCL-26	BS CL 09	66
	CYNOM-K1	Monkey Cynomolgus Skin	ECACCˆ 90071809	BS CL 221	25
	CV-1	African Green Monkey Kidney	ATCC CCL-70	BS CL 23	45
	FRhK-4	Fetal RhesusMonkey Kidney	ATCC CRL-1688	BS CL 115	78
	FrP3	Fetal Rhesus Monkey Kidney	ISS*	BS CL 169	32
	LLC-MK2	Rhesus MonkeyKidney	ATCC CCL-7	BS CL 57	37
	MA-104	Fetal Monkey Kidney	University of Perugia, Italy	BS CL 61	33
	MARC-145	Fetal Monkey Kidney	MARC•	BS CL 127	25
	NCTC cl 3526	Rhesus Monkey Kidney	ATCC CCL-7.2 [derivative of LLC-MK2]	BS CL 70	283
	RC 37	Monkey Kidney	University of Parma	BS CL 189	13
	4647	Monkey Vervet Kidney	ECACC 90091902	BS CL 223	11
	4MBr-5	Macaca Mulatta Monkey Lung	ATCC CCL-208	BS CL 222	31
Control or substrate cell lines	HeLa	Human cervix epitheloid carcinoma	ATCC CCL-2	BS CL 20	105
	HEp-2	Human larinx epidermoid carcinoma	ATCC CCL-23	BS TCL 23	363
	LCP	Lamb Choroid Plexus	Reparto substrati cellulari, IZSLER	BS PRC 21	-
	MRC-5	Embryonic human lung	ATCC CCL-171	BS CL 68	20
	RK13.6	Rabbit Kidney	Labtek˜	BS CL 196	76
	Vero	African green monkey kidney	ATCC CCL-81	BS CL 86	124
	3T3BALB/c	Mouse fibroblast	ATCC CCL-6587	BS CL 82	116

°American Type Culture Collection, USA;

ˆEuropean Collection of Authenticated Cell Cultures, UK;

*IstitutoSuperiore di Sanità, Italy;

^•^Meat Animal Research Center, USA;

˜Labtek, Corsico, Milano (I).

Moreover, seven cell lines used as controls or as substrates are reported in a separate section of Table 1. MRC-5, LLC-MK2 and RK13.6 were used as substrates in adventitious agents investigation, while HEp2 and 3T3BALB/c as positive and negative controls in tumorigenicity assays. LCP were infected with Maedi-Visna virus (VIR RE RSCIC 312) and used as retrovirus positive sample.

Furthermore, MRC-5 and LLC-MK2 cell lines were selected to prepare “master” batches of three types of *Poliovirus* (see *Poliovirus* propagation section).

Cells were cryopreserved in vapor phase nitrogen until use. After thawing at 37°C, they were diluted in MEM culture medium (Sigma-Aldrich, Milan, Italy), free of antibiotics, supplemented with 4mM L-glutamine (Sigma-Aldrich) and centrifuged at 125 g for 5 minutes at 20°C, in order to remove the dimethyl sulfoxide cryoprotectant agent. Cells were stained with Trypan Blue (Sigma-Aldrich), counted and checked for viability by a Cellometer^®^ Automated Cell Counter (Nexcelom Bioscience, USA). Finally, 1x10^5^ viable cells of each cell line were seeded in a 75 cm^2^-flask and incubated at 37°C in 5% CO_2_ in the below reported culture media, enriched with 10% (v/v) of Fetal Bovine Serum (FBS; Euroclone, Milan, Italy).

BGMK, BS-C-1, CYNOM-K1, HeLa, HEp2, LCP, LLC-MK2, MA-104, MARC-145, RK13.6 and Vero cell lines were amplified in MEM, while FRhK-4, FrP3, RC 37 and 4647 cell lines in D-MEM (Sigma-Aldrich). NCTC cl 3526 cell line was maintained in NCTC 135 medium (Thermo Fisher Scientific) and CV-1 in Eagle’s basal medium in Hanks’ BSS with amino acids and vitamins (Sigma-Aldrich). 4MBr-5 were propagated in Ham’s F K12 medium(Thermo Fisher Scientific) with 2 mM L-glutamine, adjusted to contain 1.5 g/L sodium bicarbonate and supplemented with 30 ng/ml epidermal growth factor, while 3T3BALB/c and MRC-5 cells were grown in MEM supplemented with 1 mM sodium pyruvate (Sigma-Aldrich).

### Microbiological testing

All cell lines were tested for bacteria, fungi and yeast contamination. Each cell suspension was inoculated in Agar Sabouraud, Triptic Soy Agar and Brain Heart Infusion microbiological media (Sigma-Aldrich). The mixtures were incubated for 5 days at 30°C and for 3 days at 37°C, respectively, and observed daily in order to detect any contamination. *Mycoplasma* infection was evaluated using the commercial MycoSensor PCR Assay Kit (M-Medical S.r.l., Milan, Italy) according to the manufacturer's instructions.

### Virology assays

Virus contamination of each cell line was investigated by Real-Time PCR assay. HCMV (*Human Cytomegalovirus*), HIV (*Human Immunodeficiency Virus*), HSV-1 (*Herpes Simplex Virus1*), HSV-2 (*Herpes Simplex Virus2*), EBV (*Epstein-Barr Virus*), HBV (*Hepatitis B Virus*) and HCV (*Hepatitis C Virus*) were examined by artus^®^ RG PCR Kit (Qiagen, Milan, Italy), according to the manufacturer’s instructions. HHV-6 (*Human Herpesvirus 6*), HHV-7 (*Human Herpesvirus 7*), HHV-8 (*Human Herpesvirus 8*) and HPV (*Human Papilloma Virus*) were evaluated by a Real Quality PCR kit (AB AnaliticaSrl, Padova, Italy), following the manufacturer’s instructions. Finally, *Influenza virus* type A was examined using the primers M-for (5’-AGATGAGTCTTCTAACCGAGGTCG-3’), M-rev (5’-TGCAAAAACATCTTCAAGTCTCTG-3’) proposed by van de Brand and colleagues [21], the probe INF-M (5’-TET- TCAGGCCCCCTCAAAGCCGA-BHQ1-3’, [22]) and the QuantiTect Virus kit (Qiagen), according to the manufacturer’s instructions. Positive and negative controls, provided by the manufacturer, have been included in each session.

*In vitro* testing for adventitious agents was performed in compliance with the European *Pharmacopoeia* recommendations (Cell substrates for the production of vaccines for human use) [23]. Cell culture samples and cell cryolisates were investigated on MRC-5, RK13.6 and LCC-MK2 cells grown in 24-well plates for adventitious viruses with the ability to induce cytopathic effect (CPE) (co-culture and cryolisate method). Briefly, 0.1 ml of each sample was inoculated on cell monolayers and, following adsorption for 30 minutes, cells were fed with the specific growth medium containing 3% (v/v) of FBS and incubated at 37°C in 5% CO_2_. After 7 days of growth, medium was renewed, while monolayers were observed daily for CPE for 14 days. On day 14, treated LCC-MK2 cells were removed from the incubator and tested for haemadsorption of guinea pig and chicken erythrocytes. Briefly, cell monolayers were washed and duplicate wells were overlaid with 0.5 ml of 0.5% (v/v) of guinea pig and chicken erythrocytes and, after 30 minutes of incubation at room temperature, examined for adsorption. As positive control H/A/WSN/33 (VIR RE RSCIC 50) influenza virus was used.

Cell cultures were also examined for the presence of retroviruses, using the Reverse Transcriptase Assay, colorimetric kit (ROCHE, Basel, Switzerland) for the quantitative determination of the viral Reverse Transcriptase (RT) activity. According to manufacturer’s instructions, a calibration curve was prepared from HIV-1 RT included in the kit. For the lysis of the retroviruses, 40 μl of supernatant were mixed with 40 μl of Lysis Buffer. After 30 minutes of incubation at room temperature, 20 μl of the reaction mixture were added to each reaction and HIV-1 RT standard tube. Finally, samples were incubated at 37°C for 15 h. Samples and HIV-1 RT dilutions were transferred into the wells of the MP modules and incubated for 1 h at 37°C. The solution was completely removed and the strip was rinsed 5 times with 250 μl of Washing Buffer per well for 30 s. 200 μl of anti-DIG-POD working solution were added and incubated for 1 h at 37°C. The solution was completely removed and the washing steps were repeated. Finally, 200 μl of ABTS Substrate Solution were added and the plate was incubated at room temperature for 30 minutes. The absorbance was measured at 405 nm by using a Gen5 microplate reader (Biotek, Milan, Italy) and the effective RT activity was extrapolated from the standard curve.

### Species of origin

Species of origin for the considered cell lines was evaluated by isoenzyme analysis, using the Authentikit System (Innovative Chemistry, Marshfield, USA), according to the manufacturers protocol. Briefly, cells were submitted to the extraction process by dilution of cell pellet in a specific buffer included in the kit. The enzyme activity was quantified by addition, after serial steps, of the Quench-A-Zyme Reagent by spectrophotometer (BioTek). The profiles of glucose-6-phosphatedehydrogenase, lactate dehydrogenase, nucleoside phosphorylase, malate dehydrogenase, mannose phosphate isomerase peptidase B, and aspartate aminotransferase isoenzymes were evaluated by electrophoresis. Species of origin was determined by comparing the migration distance of the analyzed isoenzyme systems with the reference distances provided by the kit [24].

### *In vitro* transformation assay

All the selected cell lines were tested for tumorigenicity according to the European *Pharmacopoeia* [23]. Soft agar colony assay was performed as previously described [25]. Briefly, 1% agar noble (Becton Dickinson, NJ, USA) was mixed with 50% of 2X MEM free of antibiotics, supplemented with 20% FBS and stratified into 6-well plates (3 ml/well). After solidification at room temperature for 1 h, this layer was overlaid with 0.8 ml of 1x10^5^cells/well suspension diluted in a mixture composed by 50% MEM supplemented by 20% FBS, and 50% of 0.6% agar noble. Plates were incubated at 20°C to allow solidification and then maintained at 37°C in 5% CO_2_ for 4 weeks and inspected daily by optical microscopy. Samples were considered negative if no cell growth was observed in soft agar, while the presence of one or more multicellular aggregates was accounted as transformation evidence. HEp2 and 3T3BALB/c cells were used, respectively, as positive and negative control.

### Tumorigenic evaluation

Potential tumorigenic evolution of the cell line that resulted to be negative in soft agar assays was evaluated by *in vivo* (Nu/Nu mice) assay, according to the European *Pharmacopoeia* [23]. The experiment was approved by the ethic committee of IZSLER and performed in compliance with ethical standards, according to the Directive 2010/63/EU on the protection of animals used for scientific purposes. Positive (HEp2 cells) and negative (3T3BALB/c) controls were included. All cell culture systems were tested at the same time and this approach allowed us to use only one positive and negative control, respectively. This strategy was applied in order to reduce the number of mice included in the *in vivo* assay, according to 3Rs principles. For the same reason, the *in vivo* test was not performed on BS-C-1and CV-1 cell lines because literature data has already reported their capacity to induce tumors in laboratory animals [18, 20]. Finally, no cell lines that induced transformed colonies in soft agar medium were included in the *in vivo* assay.

For the test, 70 athymic, 30-day-old male mice (Nu/Nu genotype), received from Harlan Laboratories, were used. They were subdivided in seven groups of 10 mice each. Five groups were inoculated with FRhK-4, MA-104, CYNOM-K1, 4647 and 4MBr-5 cell lines; one group was injected with the HEp2 cell line (positive control) and, finally, a further group was inoculated with the 3T3BALB/c cell line (negative control). All animal experiments were conducted at IZSLER, Brescia. The animals were housed on sterile bedding and with water and feed *ad libitum*. Each group was injected subcutaneously with 0.2 ml of 10^7^ cell suspension of each cell line. Five mice from each group were sacrificed 20 days after the injection, while the others were observed daily for 12 weeks. At the end of the observation period, the animals were humanely euthanized (CO_2_ inhalation). A necropsy was carried out on each mouse with the aim of detecting tumors at the injection site and in other organs (regional lymph nodes, lung, brain, spleen, kidney and liver). The local area of injection, together with the organs, were collected for histological examination and stained by haematoxylin-eosin, as described by Ferrari et al. [26]. In the event of tumor formation, animals were euthanized before the end of the observation period, in order to avoid any needless pain. The test was to be considered invalid if fewer than 9 of the 10 animals injected with the HEp2 cells, used as the positive control, did not show progressively growing tumors.

### *Poliovirus* propagation

The *Poliovirus* attenuated strains LsC 2ab Sabin type 1 (VIR RE RSCIC 48), P712 Ch 2ab Sabin type 2 (VIR RE RSCIC 182) and Leon 12alb Sabin type 3 (VIR RE RSCIC 183) were gently provided by Dr. Medici (University of Parma). LsC 2ab Sabin type 1 was cultivated in MRC-5 cells, while P712 Ch 2ab Sabin type 2 and Leon 12alb Sabin type 3 in LLC-MK2. The viruses were inoculated in the selected cell line seeded in a 75 cm^2^ flask at 1 MOI with 0.5 ml of culture medium. Infected cells were incubated at 37°C in 5% CO_2_ for 60 minutes and then added to 20 ml of culture medium supplemented by 3% (v/v) FBS; finally, samples were incubated at 37°C in 5% CO_2_ for five days. Cultures were observed daily in order to detect CPE and frozen at -80°C when CPE reached 80%. Subsequently they were thawed at room temperature, centrifuged at 1,000 g for 30 minutes at 4°C, distributed in aliquots, and stored at -80°C. The infectious titers were calculated by the Reed and Müench method [27]. The infectious titres of *Poliovirus* batches were the following: *Poliovirus* LsC 2ab Sabin type 1: 10^6.24^ TCID_50_/ml; *Poliovirus* P712 Ch 2ab Sabin type 2: 10^7.24^ TCID_50_/ml; *Poliovirus* Leon 12alb Sabin type 3: 10^7.74^ TCID_50_/ml.

### Replication of *poliovirus* types

FRhK-4, CYNOM-K1, 4MBr-5 and 4647 cell lines resulted to be devoid of transformation/tumorigenic evolution and were investigated for permissiveness to *Poliovirus*. To do this, 4x10^4^ viable cells/cm^2^ were seeded in 25 cm^2^ flasks, incubated in MEM added to 10% (v/v) of FBS at 37°C in 5% CO_2_. At 80% confluence, cells were infected at 0.1 MOI. At 80% CPE, flasks were frozen at -80°C, thawed, centrifuged at 1,540 g for 20 minutes at 4°C and supernatant was distributed into aliquots and used to evaluate the infectious titre, according to Reed and Müench formula [27].

For virus titration, each cell line was seeded in 96-well plastic plates and incubated at 37°C in 5% CO_2_ for 24 hours. The virus suspensions, collected from the different cell cultures, were diluted from 10^−1^ to 10^−8^ in culture medium and each dilution was inoculated into the corresponding cell line. Five wells were inoculated with each dilution at a volume of 100 μl/ well. Control cells were added with culture medium. The plates were incubated at 37°C, 5% CO_2_ for 60 minutes and then each plate was added to 100 μl/well of culture medium supplemented by 3% (v/v) FBS and incubated at 37°C, 5% CO_2_ for 7 days. Cell cultures were checked daily for CPE and the infectious titers were evaluated at the end of the observation period. In parallel sessions, *Poliovirus* infection has been performed on Vero and HeLa cells, as reference lines for the production of *Poliovirus*, and the obtained infectious titers were compared. These tests were performed in triplicate and the mean infectious titers were calculated.

### Statistical analysis

Differences between datasets of *Poliovirus* infectious titers were checked by one-way ANOVA, followed by a Dunn's post-hoc test for multiple comparisons. The significance threshold was set at P<0.05 (Prism 5, GraphPad Software).

## Results

### Microbiological testing and virology assays

The cell lines tested proved to be free of microbial and mycoplasma contamination. No virus, including adventitious agents and retroviruses, has been detected in the selected cell lines. Results are reported in Table 2.

**Table 2:** Cell line quality and tumorigenicity parameters.

Cell line	Bacterial and mycoplasma contamination	Viral contamination	*In vitro* tranformation ability	*In vivo* tumorigenicity
**BGMK**	**-**	**-**	+	nd
**BS-C-1**	**-**	**-**	**-**	+°
**CYNOM-K1**	**-**	**-**	**-**	**-**
**CV-1**	**-**	**-**	**-**	+°
**FRhK-4**	**-**	**-**	**-**	**-**
**FrP3**	**-**	**-**	+	nd
**LLC-MK2**	**-**	**-**	+	nd
**MA-104**	**-**	**-**	dubious	**-**
**MARC-145**	**-**	**-**	+	nd
**NCTC cl 3526**	**-**	**-**	+	nd
**RC 37**	**-**	**-**	+	nd
**4647**	**-**	**-**	**-**	**-**
**4MBr-5**	**-**	**-**	**-**	**-**

nd: not done; + and - symbols indicate presence and absence of contaminating agents or transformation/tumorigenic ability;

° reported by Furesz et al. [18] and Johnson et al.[20]

### Species of origin

The isoenzyme test confirmed the monkey origin of all cell lines selected for the study, whereas the MRC-5 proved to be of human derivation as expected. No cross-contamination was detected.

### *In vitro* transformation assay

**Fig 1:**
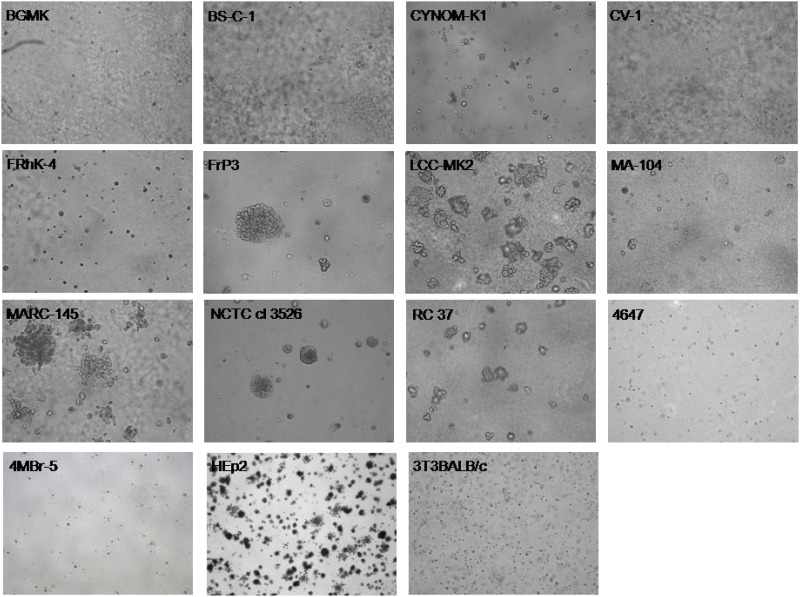
Soft agar colony assay results. Representative captures of *in vitro* transformation assay results are reported for each investigated cell line.

Cell lines reported in the higher section of Table 1 were investigated for their *in vitro* transformation ability. The results of the investigation showed that BGMK, FrP3, LLC-MK2, MARC-145, NCTC cl 3526 and RC 37cell lines induced transformed colonies in soft agar medium. These colonies began to appear at about 7 days after seeding (Fig 1) and then they increased gradually in number and size. This behavior was similar to that observed for the HEp2 cell line, used as positive control. In contrast, no transformed colonies were observed for the negative control 3T3BALB/c cell line, as well as BS-C-1, CYNOM-K1, CV-1, FRhK-4, 4647, and 4MBr-5 cell lines. All these samples remained negative until the end of the experiment (day 30). Results obtained from MA-104 cells were not clear, since cellular aggregates were detected, but their features were different from those observed in positive samples (Fig 1).

### Tumorigenic evaluation and histology

**Fig 2:**
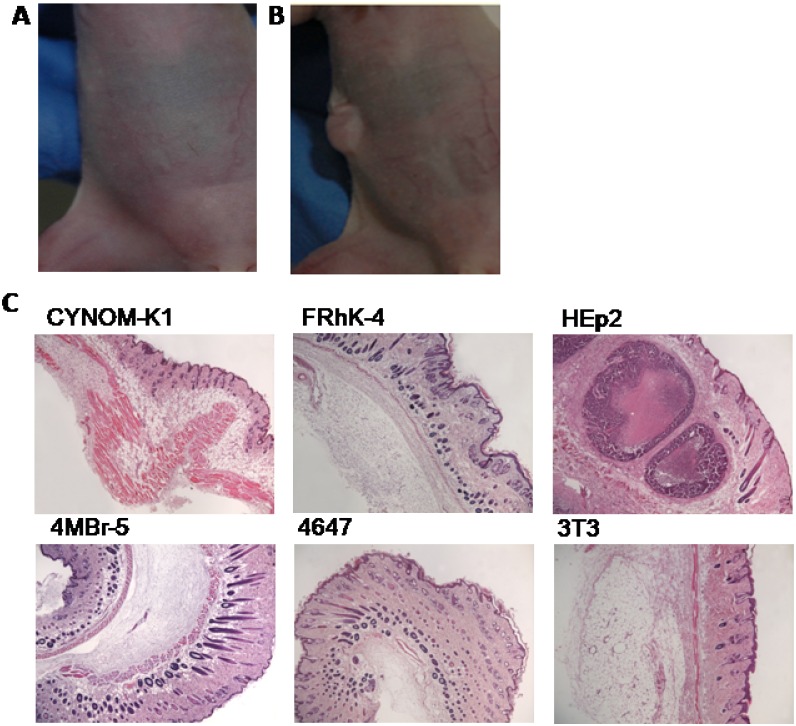
Tumorigenic evaluation and histology. Representative captures of the absence (A) and presence (B) of a nodular lesion, localized at the site of injection of cells, in treated nude mice. In Panel C histological sections derived from cell-treated mice are reported.

Cell lines that did not display transformed colonies *in vitro* were applied for further investigations to test *in vivo* tumorigenic properties. The mice injected with CYNOM-K1, FRhK-4, 4647 and 4MBr-5 cell lines did not develop any tumor formation during the observation period. The same finding was observed for the mice inoculated with the 3T3BALB/c cell line (negative control; a representative capture is reported in Fig 2A). The group injected with the positive control (HEp2) developed a hyperplastic tumor at the inoculation area, as expected. In particular, nodules were already observed about 10 days after the injection and gradually increased in size (Fig 2B). They appeared smooth, uniform and globular (10 mm Ø); later they developed a multi-globular shape and increased in size (20 mm Ø). At necropsy, a tumour was detected only at the cell injection site of the skin; no other macroscopic alterations in the other organs and tissues were detected.

At the two necropsy times (20 days and 12 weeks after the injection) no tumors were detected in skin of the *inoculum* area, lymph nodes, lungs, brain, spleen, kidneys and liver of the mice injected either with the selected cell lines under investigation or with the 3T3BALB/c cell line.

The histology carried out on tissue samples taken from mice injected with HEp2 cells showed the presence of polygonal cells in subcutaneous and dermal tissues; the nuclei of such cells were irregular in shape, with evident nucleoli (atypical mitosis); moreover, neoplastic cells were observed in the vessels. These alterations were restricted to the *inoculum* site. The tissues and organs of the animals injected with either CYNOM-K1, FRhK-4, 4MBr-5, 4647 cell lines, or those treated with the negative control 3T3BALB/c cells, did not show any inflammatory process (Fig 2C). In fact the injected cells were completely re-absorbed within a few days (in mean seven days).

Concerning MA-104 cells, the presence of a foreign body granuloma was detected at the injection *inoculum* site (data not shown). The results of the *in vitro* and *in vivo* tests are summarized in Table 2.

### Replication of *poliovirus* types

**Fig 3:**
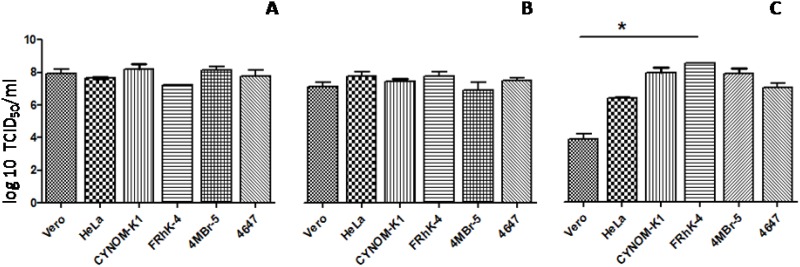
Susceptibility of cell lines to *Poliovirus*. Attenuated *Poliovirus* Sabin LsC 2ab Sabin type 1 (Panel A), P712 Ch 2ab Sabin type 2 (B) and Leon 12alb Sabin type 3 (C) infectious titers obtained on the indicated cell lines. Data are reported as TCID_50_/ml log_10_ mean ± standard error of the mean. Asterisk indicates a P value < 0.05 significance between the two data sets marked by the line.

All *Poliovirus* types replicated in all the investigated cell lines, as well as in Vero and HeLa cells, tested simultaneously as reference substrates for *Poliovirus* propagation and vaccine manufacturing. The infectious titers obtained in different assayed cells relative to attenuated Sabin strains type 1, 2 and 3, are reported in Fig 3 in Panel A, B and C, respectively. TCDI_50_/ml data are reported as log10 mean ± standard error of the mean and analysed as indicated in material and methods section. Concerning LsC 2ab Sabin type 1 and P712 Ch 2ab Sabin type 2, TCID_50_/ml infectious titers ranged between 10^7.24^ and 10^8.16^, while Leon 12alb Sabin type 3 between 10^3.83^ and 10^8.50^. They replicated into cells without showing any dissimilarity between the different substrates, exception made for the observed tendency of all investigated cell lines in being more permissive to *Poliovirus* Leon 12alb Sabin type 3 propagation than HeLa and Vero. In particular, FRhK-4 cells showed to be significantly more sensitive to Leon 12alb Sabin type 3, compared with the simian reference cell line Vero (TCID_50_/ml mean: 10^8.50^ versus 10^3.83^).

## Discussion

Cell cultures are widely used as substrates for the production of many biotechnological and biological products for human use, such as viral vaccines. Since the use of biological material may lead to potential contamination with known and unknown extraneous agents, cell substrates must be properly tested in order to avoid adverse features in patients. In this respect, the guidelines provided by the International Regulatory Agencies (European Medicines Agency, European *Pharmacopeia*, U.S. Food and drug Administration, World Health Organisation) [23, 28–30] require cell line free of any adventitious agents, eubacteria and cross-contaminants. In particular, continuous cell lines may represent a risk for the recipients of the biologicals, since they may vehicle oncogenic and viral components derived from the immortalization event (serial subcultivation of a primary cell culture of a human or animal tumor, transformation by oncogenic viruses, *in vitro* spontaneous transformation). For these reasons, cell cultivation history has to be clearly reported and transformation and tumorigenicity properties properly evaluated. Currently, the use of several tumorigenic cell lines is accepted for the manufacturing of viral vaccines, upon risk-benefit evaluation. For example, Vero cells are still used in *Poliovirus* vaccine production, although recent data showed growing evidence of tumorigenicity acquisition during serial subcultivation [16–18].

The aim of this study was to characterize different simian cell lines and assess their suitability for large-scale production. In particular, different aspects were investigated: microbiological evaluation, species of origin, potential *in vitro* and *in vivo* tumorigenic evaluation and *Poliovirus* amplification.

Microbiological results demonstrated the absence of viral and mycoplasma contamination and the isoenzyme test confirmed the monkey origin for all the analyzed cell lines.

The most crucial point regarding *in vitro* and *in vivo* tumorigenic evaluation permitted to express some interesting considerations.

Among the thrirteen cell lines investigated, only six (BS-C-1, CV-1, FRhK-4, CYNOM-K1, 4647, 4MBr-5) did not induce *in vitro* transformed colonies. Inconclusive findings were found for MA-104 cell line, since its soft-agar colonies appeared to be morphologically different from those observed in positive control.

Since the *in vitro* transformation results do not completely overlap with *in vivo* tumorigenicity ones, these cell lines were also injected into athymic mice, resulting free of any tumorigenic characteristics. In particular, CYNOM-K1, FRhK-4, 4MBr-5, 4647 cell lines did not show any evidence of macroscopic pathological tumors; necropsy was performed in order to collect sample for histological analysis. The obtained outcomes demonstrated the absence of pathological lesions either of neoplastic or inflammatory origin for all the selected cell lines.

BS-C-1 and CV-1 cell line were not assayed because already published data reported their tumorigenic features [18–20]. Among the five tested cell lines, results of the *in vivo* tumorigenicity test confirmed the data obtained from the transformation assay performed through the *in vitro* test and they both indicate no transformation features. The lack of previous studies reporting *in vivo* investigation of the tested lines allows no comparison of the results.

FRhK-4, CYNOM-K1, 4647, 4MBr-5 cell lines allowed the growth of the three viral types of attenuated Sabin *Poliovirus* as well as Vero and HeLa cells, with even superior performance in the case of Leon 12alb Sabin type 3. Since they resulted not transformed, devoid of tumorigenicity and characterized by a good replication capacity, these cells may represent alternative substrates for *Poliovirus* production to primary cell cultures from monkey kidneys, or other potentially tumorigenic cell lines, as Vero [31]. However, the continuous growth of FRhK-4 and 4647 cells, compared to the finite CYNOM-K1 cell line and the EGF-dependent 4MBr-5 [32, 33], make these cell lines more indicated for a large-scale production. Finally, another practical parameter to take in consideration is the *in vitro* growth of these cell lines. It was noticed a different trend in the doubling time of each biological substrate. As described in literature [34], Vero cells grow in 24–48 hours until a 70%-80% of confluence that represents a correct percentage for viral amplification use. During the present study, FRhK-4 and 4647 cells, as above mentioned, demonstrated a doubling time similar to Vero cells (48 hours) in comparison to CYNOM-K1 and 4MBr-5, that reach 70%-80% of confluence after 72 or more hours of incubation.

These results outlined the importance of a deep and constant monitoring of biological substrates, in order to highlight all potential risks related to the uncontrolled serial amplification. Cell batches for biological manufacturing should be maintained at established *in vitro* passages and properly tested according to the good laboratory practice and international guidelines.

## References

[1] Landsteiner K, Popper E. Mikroscopische Präparate von einem menschlichen und zwei Affentückermarker. Wein klin. Wschr. 1908;21: 1930.

[2] Racaniello VR, Baltimore D. Molecular Cloning of Poliovirus cDNA and Determination of the Complete Nucleotide Sequence of the Viral Genome in Proceedings of the National Academy of Sciences. 1981;78: 4887–4891. 10.1073/pnas.78.8.4887 PMC320284 6272282

[3] Bodian D, Morgan IM, Howe HA. Differentiation of types of poliomyelitis viruses; the grouping of 14 strains into three basic immunological types. Am J Hyg. 1949;49: 234–245. 18113220

[4] Pearce JM. Salk and Sabin: Poliomyelitis immunization. J Neurol Neurosurg Psychiatry. 2004;75:1552 10.1136/jnnp.2003.028530 15489385 PMC1738787

[5] Melnick JL. Current status of poliovirus infections. Clin Microbiol Rev. 1996;9: 293–300. 8809461 10.1128/cmr.9.3.293 PMC172894

[6] Kanda Y, Melnick JL. *In vitro* differentiation of virulent and attenuated polioviruses by their growth characteristics on MS cells. J Exp Med. 1959;109: 9–24. 13611161 10.1084/jem.109.1.9 PMC2136936

[7] Enders JF, Weller TH, Robbins FC. Cultivation of the Lansing Strain of Poliomyelitis Virus in Cultures of Various Human Embryonic Tissues. Science. 1949;109: 85–87. 10.1126/science.109.2822.85 17794160

[8] van Wezel AL. Growth of cell-strains and primary cells on micro-carriers in homogeneous culture. Nature. 1967;216: 64–65. 4292963 10.1038/216064a0

[9] Dahling DR, Wright BA. Optimization of the BGM cell line culture and viral assay procedures for monitoring viruses in the environment. Appl. Envir. Microbiol. 1986; 790–812. 10.1128/aem.51.4.790-812.1986 PMC238965 3010860

[10] Montagnon BJ, Fanget B, Nicolas AJ. The large-scale cultivation of VERO cells in micro-carrier culture for virus vaccine production. Preliminary results for killed poliovirus vaccine. Dev Biol Stand.1981;47: 55–64. 6785126

[11] Montagnon BJ, Fanget B, Vincent-Falquet JC. Industrial-scale production of inactivated poliovirus vaccine prepared by culture of Vero cells on microcarrier. Rev Infect Dis. 1984;6: S341–344. 6740071 10.1093/clinids/6.supplement_2.s341

[12] Butler M, Burgener A, Patrick M, Berry M, Moffatt D, Huzel N, et al Application of a serum-free medium for the growth of Vero cells and the production of Reovirus. Biotechnol Prog. 2000;16: 854–858. 10.1021/bp000110 11027181

[13] Paillet C, Forno G, Kratje R, Etcheverrigaray M. Suspension-Vero cell cultures as a platform for viral vaccine production. Vaccine. 2009;27: 6464–6467. 10.1016/j.vaccine.2009.06.020 19559123

[14] Frazzati-Gallina NM, Paoli RL, Mourão-Fuches RM, Jorge SA, Pereira CA. Higher production of rabies virus in serum-free media cell cultures on microcarriers. J Biotechnol. 2001;92: 67–72. 11604174 10.1016/s0168-1656(01)00362-5

[15] Milanesi E, Ajmone-Marsan P, Bignotti E, Losio MN, Bernardi J, Chegdani F, et al Molecular detection of cell line cross-contaminations using amplified fragment length polymorphism DNA fingerprinting technology. *In vitro* Cell Dev Biol Anim. 2003; 39: 124–130. 10.1007/s11626-003-0006-z 14505435

[16] Zhang DL, Liu SG, Yan LF, Li LJ, Huang GS, Fang FD, et al Carcinogenesis or tumorigenicity testing of animal cell lines for vaccine preparation by colony formation on soft agar and by agglutination under plant lectins. Cell Biol Int.2001;25: 997–1002. 10.1006/cbir.2001.0745 11589616

[17] Levenbook IS, Petricciani JC, Elisberg BL (1984) Tumorigenicity of Vero cells. J Biol Stand 12:391–398. 6526826 10.1016/s0092-1157(84)80063-3

[18] Furesz J, Fanok A, Contreras G, Becker B. Tumorigenicity testing of various cell substrates for production of biological. Dev Biol Stand. 1989;70:233–243. 2759351

[19] Contreras G, Bather R, Furesz J, Becker BC. Activation of metastatic potential in African green monkey kidney cell lines by prolonged *in vitro* culture. *In vitro* Cell Dev Biol. 1985; 21: 649–652. 4066602 10.1007/BF02623298

[20] Johnson JB, Noguchi PD, Browne WC, Petricciani JC. Tumorigenicity of continuous monkey cell lines in *in vivo* and *in vitro* system. Develop Biol Standard. 1981;50: 27–35. 6804291

[21] van den Brand J, Stittelaar KJ, van Amerongen G, van de Bildt MW, Leijten LM, Kuiken T, …, Osterhaus A. Experimental Pandemic (H1N1) 2009 Virus Infection of Cats. Emerging Infectious Diseases. 2010;16(11): 1745–1747. 10.3201/eid1611.100845 21029533 PMC3294532

[22] Huber I, Campe H, Sebah D, Hartberger C, Konrad R, Bayer M, Busch U, Sing A. A multiplex one-step real-time RT-PCR assay for influenza surveillance. Euro Surveill. 2011;16(7): pii: 19798. 21345319

[23] Cell substrates for the production of vaccines for human use In: European Pharmacopoeia. 8thedition; 2014 Chapter 5.2.3 pp. 582–585.

[24] Hull RN, Cherry WR, Tritch OJ. Growth characteristics of monkey kidney cell strains LLC-MKT, LLC-MK2, and LLC-MK(nctc-3196) and their utility in virus research. J Exp Med. 1962;115: 903–918. 14449901 10.1084/jem.115.5.903 PMC2137539

[25] MacPherson I, Montagnier L. Agar suspension culture for the selective assay of cells transformed by polyoma virus. Virology.1964;23: 291–294. 14187925 10.1016/0042-6822(64)90301-0

[26] Ferrari M, Scalvini A, Losio MN, Corradi A, Soncini M, Bignotti E, et al Establishment and characterization of two new pig cell lines for use in virological diagnostic laboratories. J Virol Methods. 2003;107: 205–212. 12505635 10.1016/s0166-0934(02)00236-7

[27] Reed LJ, Müench H. A simple method of estimating fifty percent endpoints. The American Journal of Hygiene. 1938; 27: 493–497.

[28] Quality of Biotechnological Products: Derivation and Characterisation of Cell Substrates used for Production of Biotechnological/Biological products. European Medicines Agency (EMA).CPMP/ICH/294/95, 1998. ICH Topic Q5D 9737399

[29] Characterization and Qualification of Cell Substrates and other Biological Materials used in the production of viral vaccines for infectious diseases. Food and Drug Administration (FDA).Guidance for Industry Indications. U.S. Department of Health and Human Services FDA Center for Biologics Evaluation and Research, February 2010.

[30] Recommendations for the evaluation of animal cell cultures as substrates for the manufacture of biological medicinal products and for the characterization of cell banks. World Health Organisation (WHO). Replacement of Annex 1 of WHO Technical Report Series n° 878.

[31] Manohar M, Orrison B, Peden K, Lewis AM Jr. Assessing the tumorigenic phenotype of VERO cells in adult and newborn nude mice. Biologicals. 2008;36: 65–72. 10.1016/j.biologicals.2007.06.002 17933552

[32] Wallace RE, Vasington PJ, Petricciani JC, Hopps HE, Lorenz DE, Kadanka Z. Development and characterization of cell lines from subhuman primates. In Vitro;8: 333–341 4633070 10.1007/BF02619057

[33] Caputo JL, Hay RJ, Williams CD. The isolation and properties of an epithelial cell strain from rhesus monkey bronchus. In Vitro. 1979;15: 222–223.

[34] Li W, Han Y, Yang H, Wang G, Lan R, Wang JY. Preparation of microcarriers based on zein and their application in cell culture. Materials science and engineering. C, Materials for biological applications;58: 863–869. 10.1016/j.msec.2015.09.045 26478381

